# Innate Lymphoid Cells: Role in Immune Regulation and Cancer

**DOI:** 10.3390/cancers14092071

**Published:** 2022-04-21

**Authors:** Douglas C. Chung, Nicolas Jacquelot, Maryam Ghaedi, Kathrin Warner, Pamela S. Ohashi

**Affiliations:** 1 Department of Immunology, University of Toronto, Toronto, ON M5S 1A8, Canada; 2 Princess Margaret Cancer Centre, University Health Network, Toronto, ON M5G 2M9, Canada; nicolas.jacquelot@uhnresearch.ca (N.J.); maryam.ghaedi@uhnresearch.ca (M.G.); kathrin.warner@uhnresearch.ca (K.W.)

**Keywords:** immune regulation, cancer, innate lymphoid cells, natural killer cells, tumour microenvironment, ILCregs, T cells, Treg, adaptive immunity, immune suppression, ovarian carcinoma

## Abstract

**Simple Summary:**

Innate lymphoid cells (ILCs) are an emerging family of effector cells known to play a major role in innate defenses against pathogens, lymphoid organogenesis, tissue repair, and homeostasis. They are positioned strategically within tissues to provide the first line of defence and shape the ensuing adaptive immune cell response. Recent evidence suggests that ILCs contribute to immune regulation in different diseases, including cancer, and can have significant impact on disease outcome. In this review, we highlight the immunosuppressive roles of ILCs in cancer that inhibit effective immune surveillance and anti-tumour response.

**Abstract:**

Immune regulation is composed of a complex network of cellular and molecular pathways that regulate the immune system and prevent tissue damage. It is increasingly clear that innate lymphoid cells (ILCs) are also armed with immunosuppressive capacities similar to well-known immune regulatory cells (i.e., regulatory T cells). In cancer, immunoregulatory ILCs have been shown to inhibit anti-tumour immune response through various mechanisms including: (a) direct suppression of anti-tumour T cells or NK cells, (b) inhibiting T-cell priming, and (c) promoting other immunoregulatory cells. To provide a framework of understanding the role of immunosuppressive ILCs in the context of cancer, we first outline a brief history and challenges related to defining immunosuppressive ILCs. Furthermore, we focus on the mechanisms of ILCs in suppressing anti-tumour immunity and consequentially promoting tumour progression.

## 1. Introduction

**Figure 1:**
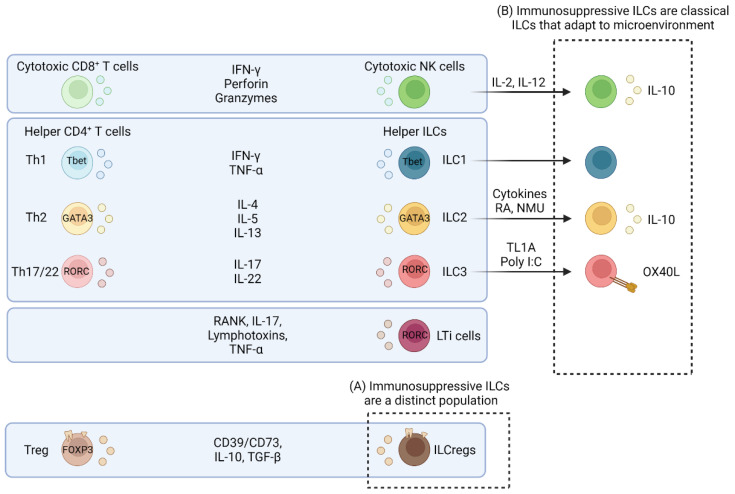
ILCs, including ILCregs, are classified based on functional similarities to T cell counterparts. The conventional members of ILCs (i.e., NK cells, ILC1s, ILC2s, ILC3s) were categorized based on their similarities in expression of transcription factors and cytokines to T cells (i.e., CD8^+^ T cells, Th1, Th2, and Th17 CD4^+^ helper T cells). Based on our current understanding, immunosuppressive ILCs could be added to the current classification as (**A**) a distinct population of cells (i.e., ILCregs) that share immunosuppressive features with Tregs or (**B**) as an extension of conventional ILCs that adopt suppressive features (e.g., IL-10, OX40L) following stimulation with the appropriate microenvironmental signals.

Innate lymphoid cells (ILCs) represent a heterogenous population of cells that mostly reside within non-lymphoid peripheral tissues. The family of ILCs is currently made up of five subpopulations, namely natural killer (NK) cells, ILC1s, ILC2s, ILC3s, and lymphoid tissue inducer (LTi) cells, and broadly resemble cytokine secretion and transcription factor profiles of T cell subsets (Figure 1) [1,2]. ILCs are known to adopt new functions and phenotypes in response to fluctuating environmental cues through a process known as functional plasticity [3,4,5,6]. Tissue-resident properties of ILCs position them well to respond rapidly to tissue-specific stimuli and orchestrate innate and adaptive immunity in response to infections [7,8,9]. In addition to the classical roles of ILCs in promoting inflammatory response, there has been increasing recognition that ILCs also have immunoregulatory properties in various diseases including cancer [10,11,12,13]. In this review, we provide a framework for the potential origins and properties of immunoregulatory ILCs and highlight their inhibitory role in cancer. 

## 2. Regulatory Innate Lymphoid Cells—Emerging Players in Immune Regulation

### 2.1. Early Discoveries of Immunosuppressive ILCs 

NK cells were the first of the ILC family members to be discovered, initially described in 1975 as cytolytic killers that targeted transformed cancer cells and virally infected cells [14,15]. Since 1985, studies were published implicating the role of NK cells as immunological suppressors. NK cells were reported to inhibit hematopoiesis [16,17,18] and B cell proliferation and function [19,20,21,22,23,24,25,26]. Most notably, NK depletion increased autoantibody-secreting B cells in a *lpr* mutant mouse model of autoimmunity [26]. Around the same time, several studies reported that NK cells are able to kill autologous antigen-presenting cells (APCs) in vitro, indirectly inhibiting T cell function [27,28,29,30,31,32]. This early literature established the potential of NK cells in negatively regulating immune function. 

In 1998, Fort et al. found that NK cell depletion exacerbated a T-cell-dependent model of colitis [33]. Studies using murine cytomegalovirus (MCMV) infection models found that NK depletion increased viral titers and enhanced viral-specific T cell responses [34,35,36,37]. However, the immunoregulatory contributions of NK cells in MCMV infections were hard to distinguish from their direct role in preventing early viral replication [38]. In contrast, NK cells are dispensable in the direct clearance of lymphocytic choriomeningitis virus (LCMV) early in infection [38,39]. Studies have shown that NK cells from LCMV-infected mice suppressed CD8^+^ and CD4^+^ T cell mediated viral clearance through an activating receptor natural killer group 2, member D (NKG2D) and cytolytic molecule perforin [40,41]. Additionally, NK1.1^+^ cells prevented T-cell-mediated autoimmunity in the salivary glands and liver of LCMV-infected mice [42,43,44]. It is known that NK1.1^+^ cells in the salivary glands, liver, uterus, and other tissues make up a mixture of ILC1s and NK cells [45,46,47]. Due to the lack of technologies to selectively deplete ILC1s or NK cells in vivo, it remains to be defined which of these subsets play an immunosuppressive role in chronic viral infections. Collectively these studies highlight the role of NK-like cells in direct suppression of viral-specific T cells. Additionally, several groups have described the roles of NK-like immunoregulatory cells in autoimmunity [48,49,50,51,52,53,54], graft-versus-host disease (GVHD) [55,56], and other infectious diseases [57]. Since then, ILCs with immunosuppressive properties have been identified in diverse contexts and mechanisms of suppression [10,12,58]. For example, immunosuppressive ILC2s reduced allergen-induced lung inflammation [59], enhanced islet allograft survival [60], and reduced joint inflammation in a model of arthritis [61]. Furthermore, immunoregulatory ILC3s are involved in reducing GVHD [62] and shape intestinal homeostasis [63,64,65].

In 2017, two groups have identified immunosuppressive ILCs that do not fit the criteria of any conventional ILC subset as ILCregs [66,67]. In ex vivo tumour-infiltrating lymphocyte (TIL) cultures from patients with high-grade serous ovarian carcinoma (HGSOC), our group identified CD56^+^ ILCregs that suppressed autologous T cells in a contact-dependent manner in vitro. CD56^+^ ILCreg-mediated suppression of T cells was partially dependent on natural killer cell p46-related protein (NKp46). Moreover, CD56^+^ ILCregs were not distinctly NK cells because they expressed cytokines typical of other ILC populations (i.e., IL-9 and IL-22). Overall, our findings suggest that CD56^+^ ILCregs are a suppressive population distinct from conventional ILCs. Similar populations of suppressive CD56^+^ ILCs have also been reported by other groups [68,69,70,71,72]. In addition, a study by Wang et al. identified an IL-10^+^ ILCreg population that did not express other conventional ILC markers and suppressed ILC1s and ILC3s to prevent intestinal inflammation [67]. However, this study was met with controversy since Bando et al. were unable to observe a substantial IL-10 secreting population [73] using the same intestinal inflammation model [67]. 

Taken together, these studies provide evidence of a heterogenic population of immunosuppressive ILCs. In this review, we propose a model of immunosuppressive ILCs which provides two separate, but not mutually exclusive, paths of origin and may explain the heterogeneity and challenge in providing a unified definition of immunomodulatory ILCs (Figure 1). It is possible that there is a distinct regulatory subset based on the findings that the mechanisms of inhibition are similar to mechanisms used by Treg subsets. In this case, immunosuppressive ILCs could be clustered within the category of ILCregs (Figure 1A). However, there are currently no lineage-specific transcription factors that define ILCregs, which prompts the question of whether these cells are indeed a distinct ILC subset. On the other hand, immunosuppressive ILCs may be a product of conventional ILCs that adopt immunosuppressive programs in response to tissue-specific signals (Figure 1B). This is supported by studies that have shown that conventional ILC subsets could be induced to express immunosuppressive molecules in vitro after exposure to cytokines or other soluble mediators [59,64,73,74,75,76]. In the next sections, we will briefly review evidence supporting both models. 

### 2.2. Are Immunosuppressive ILCs a Distinct Population with Similar Mechanisms as Tregs?

**Figure 2:**
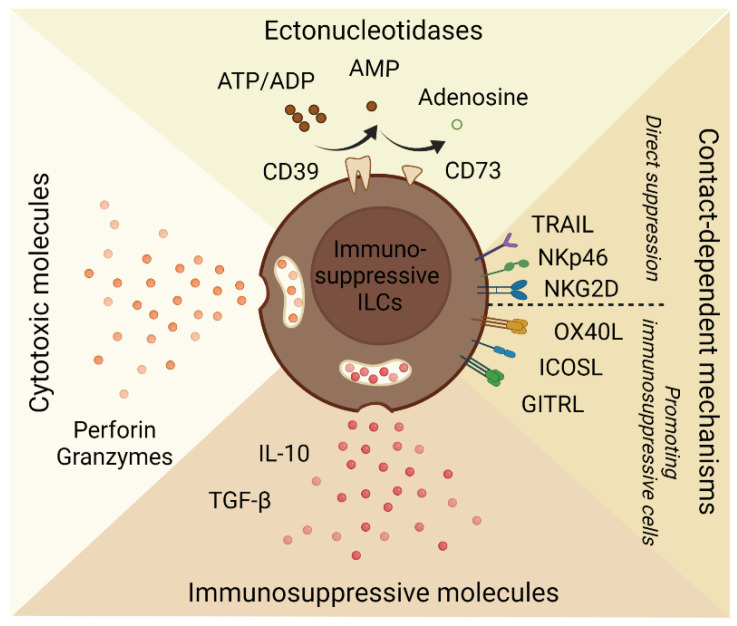
Mechanisms of immunosuppressive ILCs. Immunosuppressive ILCs have been shown to suppress immune responses through ectonucleotidases (e.g., CD39, CD73), killing of immune cells through cytotoxic molecules (e.g., perforins, granzymes) or contact-dependent mechanisms (e.g., TRAIL, NKp46, NKG2D), and soluble mediators (e.g., IL-10, TGF-β). Immunosuppressive ILCs can promote other suppressor cells through expression of OX40L, ICOSL, and GITRL.

Tregs were initially classified as a distinct population on the basis of their suppressive functions [77,78]. Thus, to further understand the concept of ILCregs, it is helpful to compare their immunosuppressive functions (Figure 2) with Tregs. Tregs play an essential role in maintaining immune tolerance and preventing autoimmunity in various contexts [79]. Tregs are characterized by forkhead box protein P3 (FOXP3) which is essential to their development, stability, and suppressive activity [80,81,82,83]. Although there is currently no evidence that immunosuppressive ILCs express FOXP3, they share functional similarities and mechanisms with Tregs. For example, Tregs can directly suppress T cells through cytolysis, cytokine-mediators (e.g., IL-10, TGF-β), and ectonucleotidases (e.g., CD39, CD73) [84]. As mentioned above, ILCs have been shown to suppress T cells through perforin- and NKG2D-mediated killing [40,41,54,85] and secretion of IL-10 [58,67]. Furthermore, CD39 and CD73 expression on human RORγt^+^ ILC3s were essential in suppressing autologous T cell proliferation in vitro [62]. CD73 on ILC2s also suppressed NK cell activation in vitro [86]. 

Tregs and immunosuppressive ILCs both inhibit APC priming of T cells. Tregs preferentially aggregate around APCs in the lymph node and attenuate APC-T cell synapses to prevent priming [87,88,89,90]. Tregs express cytotoxic T-lymphocyte-associated protein 4 (CTLA-4) which binds to and removes CD80/CD86 on APCs, preventing co-stimulatory signaling through CD28 on T cells [91,92]. Additionally, Tregs suppress APCs through IL-10 [93,94] and can directly kill them through granzyme and perforin [95]. As discussed in later sections, NK cells also directly kill APCs in the lymph nodes to prevent T cell priming [96,97,98,99]. Moreover, Blois et al. proposed that decidual NK cells secrete IL-10 to suppress APCs and prevent fetal rejection during pregnancy [100]. Finally, FOXP3^+^ follicular Tregs suppress T-follicular helper (Tfh) CD4^+^ T-cell-induced B cell antibody production in vitro and in vivo [101,102]. Similarly, O’Conner et al. found that follicular ILCs are present in human tonsillar and lymph node germinal centers. These follicular ILCs expressed TGF-β and suppressed Tfh-supported IgG expression by B cells in vitro [103]. Overall, immunosuppressive ILCs and Tregs share common mechanisms of immune suppression. These similarities mirror how other conventional ILC subsets were stratified depending on their similarities to CD4^+^ and CD8^+^ T cell subsets (Figure 1), supporting the idea of classifying ILCregs as a distinct population of ILCs. However, if the history of Tregs is of any guide, the discovery of an ILCreg-defining surface molecule or transcription factor and fate-mapping experiments will be essential to further understand the biology of this immunosuppressive ILC subset. 

### 2.3. Are ILCregs a Variation of Conventional ILCs That Were Turned into a Suppressive State?

#### 2.3.1. ILCs Adopt Immunosuppressive Programs in Response to Environmental Signals

ILCs are heavily conditioned by the microenvironment and could influence innate and adaptive immunity in response to various tissue-specific signals [8]. In contrast to the proponent that ILCregs are a distinct subset, several studies have demonstrated that conventional ILCs adopt immunosuppressive functions in response to environmental cues. As mentioned above, IL-10 expressing ILCs have been shown to play immunosuppressive roles in various contexts. In 2017, Seehus and colleagues found that ILC2s (CD90.2^+^ Lin^−^ ST2^+^) expressed IL-10 in models of lung inflammation [76]. Moreover, ILC2s suppressed proliferation of CD4^+^ T cells in an IL-10 dependent manner in vitro [59]. Interestingly, IL-10 expression can be induced from mouse or human conventional ILC2s after exposure to a combination of IL-2, IL-33, and retinoic acid (RA) in vitro [59,76]. Bando et al. showed that intestinal ILC2s can express IL-10 after exposure to IL-2, IL-4, IL-10, IL-27, or neuromedin U (NMU) [73]. Similarly, IL-2 and IL-12 can induce human or murine NK cells to express IL-10 [74,75]. These studies suggest that conventional NK cells and ILC2s can adopt IL-10 expression in response to cytokine signals. 

ILCs can promote the expansion of Tregs through cell-to-cell contact mechanisms. For example, ILC2s promoted the expansion of Tregs through OX40L in lungs of fungal or helminth infectious models [104]. OX40L can be induced on ILC2s with IL-33 in vivo. Deng et al. also demonstrated that OX40L derived from ILC3s was important for maintaining Treg homeostasis in the intestine [64]. Furthermore, intestinal Lin^−^RORγt^+^ ILC3s could be induced to express OX40L in response to tumour necrosis factor-like cytokine 1A (TL1A) or Poly I:C in vitro in a STAT5 dependent manner. These studies suggest that conventional ILC2s and ILC3s may adopt Treg-like immunosuppressive programs in response to specific cytokine cues. 

#### 2.3.2. ILC-Mediated Suppression by Cytolysis May Be Governed by Target Cell Features

Immunosuppressive outcomes by conventional ILCs may be governed by the expression of activation and inhibitory ligands on target cells. For example, NK cells can kill immature APCs to promote inflammatory responses [105,106], but also kill mature APCs to suppress T-cell-mediated chronic viral clearance [36,107]. Mature APCs were shown to be less susceptible to killing by NK cells compared to immature APCs due to enhanced expression of inhibitory human leukocyte antigen (HLA) class I molecule [108]. Interestingly, APCs from human immunodeficiency virus (HIV)-infected individuals were more susceptible to ILC-mediated cytolysis through the upregulation of MIC-A/B, (a NKG2D ligand), after exposure to IL-10 [107]. Moreover, the expression of nectin-2 (CD112) and poliovirus receptor (CD155) on APCs promoted their lysis by NK cells [109]. These studies suggest that immunosuppressive properties of NK cells may be governed by the expression of ligands on target APCs. 

Similarly, the susceptibility of T cells to ILC-mediated killing is dependent on the expression profile of various ligands. In LCMV infections, type I interferon (IFN) protected anti-viral CD8^+^ T cells from NK-mediated cytotoxicity [110,111] by upregulation of inhibitory ligand Qa-1b [112]. Moreover, inhibitory ligands CD48 [41] and major histocompatibility complex (MHC) I [113,114] expression on T cells reduced their susceptibility to killing by 2B4 and KIR expressing NK cells. Finally, activated, but not resting, T cells expressed NKG2D ligands including MIC-A/B, increasing their susceptibility to NK cell-mediated killing [85,115,116,117]. Overall, these findings suggests that the intrinsic properties of target cells (i.e., activation/maturation status, and the expression of various ligands) dictate whether or not they are susceptible to NK-mediated killing. It is plausible that these suppressive ILCs are indistinguishable from conventional NK cells, and that the ‘immunosuppressive’ phenomena observed may be dictated by changes in activation and inhibitory ligands by target cells. 

## 3. ILCs have Tumour-Promoting Roles in Human and Mice 

Numerous studies in the past decade have suggested that ILCs play important roles within the tumour microenvironment (TME). Since others have extensively reviewed anti-tumour roles of ILCs [13,118,119], we will focus on the immunoregulatory and tumour-promoting potential of ILCs in cancer (Table 1). Several studies have found that certain ILC subsets were associated with poor clinical outcomes in patients with breast [120,121,122], colorectal [123], esophageal squamous cell [124], kidney [125], melanoma [126], lung [127,128,129], sarcoma [71], prostate [130], and pancreatic [131] carcinomas. NK depletion in a mouse model of fibrosarcoma led to increased overall survival and increased T cell memory response to secondary tumour challenge [97]. ILC2 depletion using transgenic mice also reduced tumour volume in mice implanted with MC38 tumour cell line [132], and increased the number of lung nodules in a lung metastasis model [133]. Adoptive transfer of RORγt^+^ ILC3s increased the number of tumour nodules in an IL-23-expressing liver cancer model [134]. Overall, these studies in humans and mice suggest that ILCs have tumour-promoting roles in certain contexts. 

## 4. Immunosuppressive Roles of ILCs in Cancer

### 4.1. ILCs Directly Suppress Cytolytic Anti-Tumour Immune Cells

#### 4.1.1. Direct Killing 

**Figure 3:**
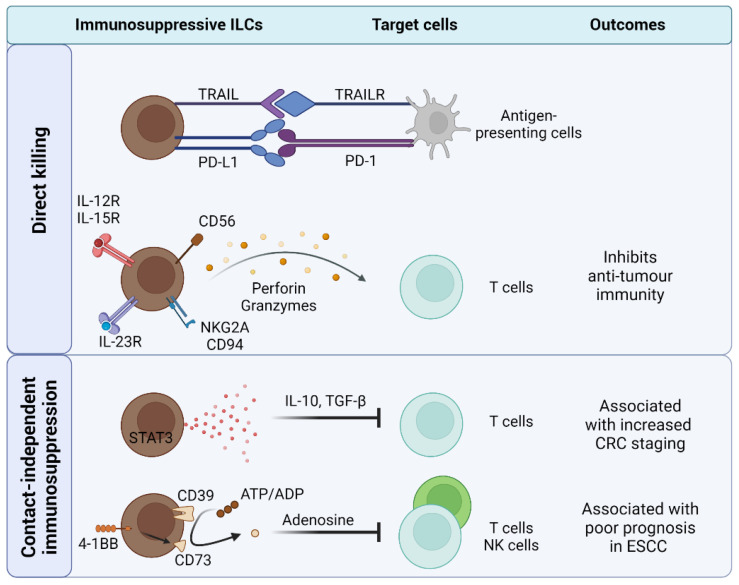
ILCs suppress anti-tumour immunity through contact-dependent and contact-independent mechanisms. ILCs play active roles in suppressing T-cell-mediated clearance of tumours through direct-killing of T cells using perforin and granzymes, production of immunoregulatory molecules (e.g., IL-10 and TGF-β), and conversion of ATP into suppressive adenosine through ectoenzymes (e.g., CD39, CD73). Furthermore, ILCs directly kill antigen-presenting cells (APCs) preventing them from cross-priming CD8^+^ T cells in the lymph nodes.

Direct killing of T cells is a key mechanism of ILC-mediated immune regulation as mentioned above. In the context of hepatocellular carcinoma (HCC), Liu et al. found that murine NKp46^−^ RORγt^+^ ILC3s reduced proliferation and induced apoptosis of CD8^+^ T cells, but not CD4^+^ T cells, in vitro [134]. T cell killing by ILC3s was not detected in a transwell system, suggesting that this mechanism was dependent on cell-to-cell contact. Interestingly, intratumoural ILC3s were transdifferentiated from ILC1s in the presence of IL-23 within the TME, suggesting that the immunosuppressive ILCs may have originated from ILC1s. Similarly, Koh et al. found that tumour cells from lung squamous cell carcinoma (LSCC) induced conversion of ILC1s to ILC3s through IL-23, and patients with high proportions of ILC3s were associated with poor progression-free survival (PFS) [128]. Overall, these findings suggest that ILC3s may suppress anti-tumour immunity through direct killing of CD8^+^ T cells (Figure 3).

It is interesting to highlight that ILC3s are selective in their target cells, killing CD8^+^ T cells but not CD4^+^ T cells. Mechanisms of cytolytic ILC3s are still not well understood. Cytokine-induced cytolytic ILC3s expressed common NK-cell-related markers including CD94 and CD56 [139,140], reminiscent of CD56^+^ ILCregs from ovarian TIL cultures [66]. Moreover, Raykova et al. showed that cytolytic ILC3s express NKG2A [139]. NKG2A is a receptor to Qa-1b (mouse) and HLA-E (human), and have been shown to play a role in preventing CD8^+^ T cell killing by NK cells in LCMV models [112]. More work is needed to fully elucidate the mechanisms of cytolytic ILC3s in mediating immune suppression in cancer. 

#### 4.1.2. Secreting Immunoregulatory Factors: IL-10

ILCs have been shown to secrete immunoregulatory factors that suppress anti-tumour immune response (Figure 3). IL-10 is well known for its immunoregulatory properties and is associated with poor prognosis in certain tumour contexts [141]. NK cells from the blood of breast cancer patients [142] and 4T1 mouse models [143] expressed higher levels of IL-10 compared to healthy subjects. Interestingly, Neo et al. found that primary tumours from breast or sarcoma cancer patients induced STAT3-dependent expression of IL-10 and TGF-β by NK cells in vitro [71]. This expression was selectively enriched on CD73^+^ NK cells. Furthermore, CD73^+^ NK cells suppressed CD4^+^ T cell proliferation and IFN-γ expression via IL-10, but not TGF-β. These findings parallel studies in murine cytomegalovirus (MCMV) where NK cells suppressed T-cell-mediated liver damage through IL-10 [144]. IL-10^+^ ILCregs were also found in colitis-associated CRC and were associated with increased cancer staging [123]. Further studies are needed to fully appreciate the mechanisms that control IL-10 program in intratumoural ILCs. 

#### 4.1.3. CD39 and CD73 Ectonucleotidases

CD39 and CD73 are ectonucleotidases that convert adenosine triphosphate (ATP) into adenosine monophosphate (AMP) and AMP into adenosine (ADO), respectively [145,146]. ADO has been shown to directly suppress proliferation and cytotoxic functions of T cells through its binding to adenosine receptors. Zheng et al. found that culture media of esophageal cancer cell lines can induce expression of CD39 on NK cells [124]. Concordantly, high frequency of intratumoural CD39^+^ NK cells in esophageal squamous cell carcinoma (ESCC) patients was associated with poor PFS and increased tumour invasion. 

CD73 is expressed on NK cells infiltrating breast cancer and sarcoma tumours [71]. In sarcoma tumours harboring a high NK cell gene signature, *NT5E* (encoding for CD73) gene expression was negatively associated with survival. Notably, CD73 expression in NK cells was inducible through 4-1BB/4-1BBL pathway [71]. Although, CD73^+^ NK cells suppressed CD4^+^ T cells in vitro, this was not dependent on the enzymatic activity of CD73 as neither the addition of exogenous AMP nor adenosine receptor inhibitors affected its suppressive activity. In a melanoma model transfected to express IL-33, CD90^+^ST2^+^ ILC2s expressed CD73 [86]. Bone marrow (BM)-derived ILC2s were able to inhibit NK-mediated cytotoxicity in the presence of AMP in a CD73-dependent manner. This suggests that ILCs may utilize CD73 to suppress cytolytic function in cells. Beyond NK cells, Ercolano et al. have found that other ILC subsets were also influenced by adenosine when co-cultured with melanoma cell lines [147]. Future studies are required to understand how CD39 and CD73 expression in ILCs regulate the activity of anti-tumour innate and adaptive immune cells.

### 4.2. ILCs Inhibit Antigen Presenting Cells

APCs orchestrate antigen-specific immunity by presenting antigens to T cells in secondary lymphoid organs [148]. Intratumoural APCs have been shown to migrate to tumour-draining lymph nodes (tdLN) and activate tumour-specific T cell responses [149,150,151]. NK cells inhibited APC-mediated cross-priming of tumour-specific T cells in vitro [97,98,99] and reduced frequency and maturation of CD11c^+^ APCs in the tdLN from a fibrosarcoma model [97]. In parallel, NK cell depletion increased antigen-specific CD8^+^ T cells in the periphery, delayed tumour growth, and enhanced memory response to secondary tumour rechallenge. Overall, these findings suggest that NK cells suppress APC-mediated activation of tumour-specific T cells (Figure 3).

As mentioned in earlier sections, NK cells are able to directly kill APCs to regulate adaptive immunity. TNF-related apoptosis-inducing ligand (TRAIL) exists in membrane-bound or soluble forms and binds to TRAIL receptors to induce apoptosis [152]. NK cell-derived TRAIL expression induced APC cell death in an apoptosis-dependent manner [99]. Moreover, adding agonistic antibodies to TRAIL receptor, DR5, reduced the ability of BM-derived APC to phagocytose tumour cells and to prime CD8^+^ T cells. Overall, these studies suggests that NK cells suppress T cells indirectly through the cytolysis of APCs. 

It is increasingly clear that ILCs exists not only in mucosal tissues, but also in secondary lymphoid organs such as lymph nodes (LNs) [153]. An elegant study using Kaede photoconvertible mice showed that ILCs in peripheral LNs are composed of both migratory and tissue-resident populations [154]. Landmark studies have recently shown that ILCs migrate from their tissue-of-residence to LNs during homeostatic and inflammatory conditions [155,156,157]. The ability of tissue-resident ILCs to migrate into LNs allows us to contemplate whether, and how, intratumoural ILCs may modulate immune cells, such as APCs, within the tdLNs. 

### 4.3. ILCs Recruit and Promote the Function of Immunosuppressive Cells

#### 4.3.1. Recruitment of Immunosuppressive Cells

**Figure 4:**
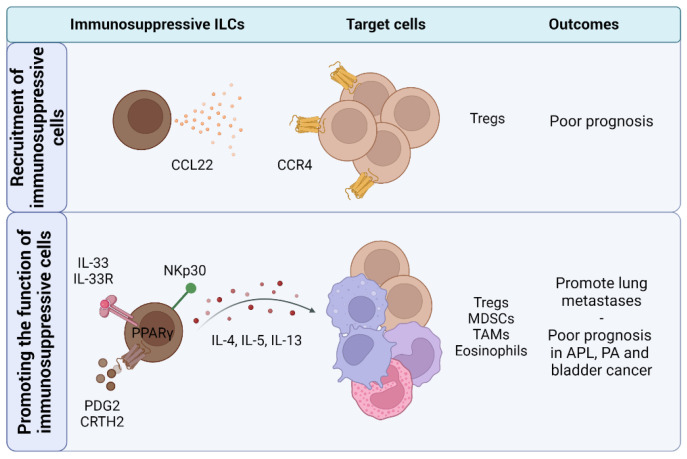
ILCs recruit and promote immunosuppressive cells in cancer. ILCs have been shown to recruit Tregs into tumours through secretion of CCL22. Furthermore, ILCs promote expansion and suppressive functions of Tregs, MDSCs, TAMs, and eosinophils through the expression of cytokines (e.g., IL-4, IL-5, IL-13). All figures were created with BioRender.com.

ILCs not only modulate immunity through secretion of cytokines but are also able to promote recruitment and infiltration of immunoregulatory cells into the TME through chemokine expression (Figure 4). In a metastatic Lewis lung carcinoma model, NK cell depletion reduced the level of C-C motif chemokine ligand (CCL) 22 [135]. Accordingly, NK cells clustered closely with Tregs within the tumour. Moreover, Mailloux et al. found that the CCL22 receptor, C-C motif chemokine receptor (CCR) 4, was primarily expressed by intratumoural Tregs and mediated recruitment of Tregs towards tumour conditioned medium in vitro. Other studies have also demonstrated that the CCL22/CCR4 pathway is involved in Treg infiltration into the TME [158,159,160]. Overall, these studies suggest that intratumoural NK cells may be involved in the active recruitment of Tregs through CCL22. It is interesting to note that CCL22 expression in NK cells can be enhanced by soluble HLA-G in vitro [161]. This provides a potential example where conventional ILCs (i.e., NK cells) may adopt specific molecular program (i.e., CCL22) in response to tissue signals, resulting in an immunosuppressive outcome (i.e., recruitment of Tregs). Moreover, recent studies have demonstrated that tumour-associated immunosuppressive ILC2s [125] and myeloid derived suppressor cells (MDSCs) [162,163] also express CCR4, prompting the need to investigate whether NK cells promote the recruitment of other immunosuppressive cells into the TME. 

Non-NK ILCs have been reported to recruit anti-tumour-promoting immune cells into tumours; however, it is not known whether they are capable of recruiting immunosuppressive cells. For example, ILCs recruit conventional dendritic cells (cDC) 1, possibly through CCL5, and enhance anti-tumour immunity [164]. Moreover, ILC2-derived granulocyte-macrophage colony-stimulating factor (GM-CSF) expression recruits anti-tumour eosinophils to limit melanoma tumour progression [165]. Overall, these findings suggest that ILCs may play a prominent role in the recruitment of immune cells into the TME. Future studies are required to understand the specific molecular switches that govern chemokine expression programs in ILCs. These studies may give rise to novel therapeutic strategies that control the ability of ILCs to recruit anti-tumour immune cells, but inhibit recruitment of immunoregulatory cells, into the TME. 

#### 4.3.2. Tregs

The frequency of Tregs is often associated with poor prognostic outcomes in patients with cervical, renal, melanoma, breast, gastric, and ovarian tumours [166,167]. Furthermore, depletion of FoxP3^+^ Tregs in preclinical studies established potent anti-tumour responses [168]. Collectively, these findings suggests that Tregs provide a formidable force in promoting immunoregulation and tumour development. 

ILCs have been recently found to promote Treg activity through the expression of surface molecules (i.e., OX40L, GITRL, ICOSL) and secretion of cytokines [61,63,104]. These include IL-2 which regulates FOXP3 expression in Tregs and induces expansion, maintenance, and stability of these cells [169,170,171]. Moreover, ILC3-derived IL-2 was essential in maintaining homeostasis of intestinal Tregs [63], Recently, Wu et al. revealed that mesenchymal stromal cells from patients with acute myeloid leukemia (AML) induced IL-5 secretion by ILC2s through prostaglandin D2 (PGD2)-CRTH2 axis in vitro [137]. IL-5 from ILC2s promoted CD4^+^CD25^+^ Treg expansion and subsequent expansion of malignant hematopoietic stem and progenitor cells. Further studies are needed to investigate the role of ILCs in promoting Treg activity within the TME. 

#### 4.3.3. MDSCs and TAMs

MDSCs are a heterogenous population of immature myeloid cells that are known for their ability to suppress antigen-specific T cells [172,173]. MDSCs have also been shown to play key roles in supporting tumour growth, inducing angiogenesis, and suppressing anti-tumour immunity in various cancers using diverse mechanisms [174,175]. In recent studies, ILC2s have been found to promote suppressive functions of MDSC through IL-13 [125,130,176]. Trabanelli et al., found that acute promyelocytic leukemia (APL) cell lines promoted IL-13 secretion by ILC2s through B7H6/NKp30 and PGD2-CRTH2 signaling pathways resulting in enhanced MDSC-mediated suppression of T cells [130]. Although the authors found that a combination treatment of antibodies that targeted PGD2, IL-13, and NKp30 increased survival in a humanized model of APL, further work with other models is needed to tease apart tumour-mediated induction of immunosuppressive ILC2s. Additionally, mouse models of breast cancer showed that ILC2-MDSC pathway promoted lung metastasis [176] and that peroxisome proliferator-activated receptor-γ (PPARγ) signalling regulated pro-tumourigenic activity and IL-13 secretion by ILC2s, revealing a new mechanism promoting MDSC activity in cancer [132].

Tumour-associated macrophages (TAMs) are cells from the myeloid lineage that express markers characteristic of mature macrophages, distinguishing them from MDSCs [177]. TAMs have been shown to have distinct immunoregulatory roles within the TME [178]. Gallazzi et al. found that peripheral NK cells from patients with prostate adenocarcinoma (PA) expressed increased levels of factors involved in monocyte recruitment and polarization (i.e., GM-CSF, CXCL1, CXCL22, CCL1, CCL2, CCL5, CCL7, CCL13, IL-10) [136]. Interestingly, culture media from tumour-associated NK cells induced THP-1 monocyte cell lines to express TAM-related genes (i.e., *Arg1*, *CXCL8*, *IL-10*). Future studies are required to demonstrate whether NK cells promote TAMs in vivo. Apart from NK cells, supernatant from PD-1^high^ ILC2s infiltrating non-small cell lung cancer (NSCLC) enhanced polarization of healthy CD14^+^ myeloid cells into M2-like macrophages through IL-4 and IL-13 [138]. Although these studies suggest that ILCs can induce a TAM-like phenotype, further work is needed to determine whether ILCs promote suppressive function of these cells. 

#### 4.3.4. Eosinophils

The role of eosinophils in cancer remains largely understudied [179]. Previous work has found that ILC2s actively facilitate eosinophil infiltration in tumours [165]. Although in this particular study, eosinophils played an anti-tumour role, they also have been found to be immunoregulatory in certain tumour models. In mice intravenously injected with both B16.F10 melanoma cell lines and IL-33, ILC2s promoted infiltration of eosinophils and increased the number of lung metastasis [133]. Interestingly, eosinophils suppressed the expression of IFN-γ and granzyme-B on NK cells. Furthermore, the authors showed that ILC2s and IL-5 promoted metastases development in breast tumour models exposed to *Aspergillus* protease-allergen (Asp). Further studies are needed to determine the cues from primary tumours and metastases that may explain context-dependent mechanisms of anti-tumour or immunoregulatory eosinophils. 

## 5. Implications of Regulatory ILCs in Ovarian Cancer and Beyond

Ovarian carcinoma is one of the most commonly occurring cancers in women, with an estimated 22,240 newly diagnosed cancers within the US in 2018 [180]. A majority of patients with ovarian carcinoma do not respond to immune checkpoint inhibitors [181,182,183,184,185], prompting the need to further understand the biology of immune regulation within the ovarian TME. It is likely that there are many immune inhibitory mechanisms at play in ovarian cancer. Previous studies have shown that Tregs [158,186,187], inhibitory soluble mediators (i.e., TGF-β, VEGF, IDO) [188,189,190], ectonucleotidases (i.e., CD73) [191,192], and immune checkpoint molecules (i.e., PD-L1, CD155, CTLA-4, B7-H3) [193,194,195,196,197] are associated with poor clinical outcomes in ovarian cancer patients. We found that ILCregs may also suppress anti-tumour immunity in ovarian cancer. In ex vivo TILs cultures, we observed that CD56^+^ ILCs inhibited the expansion of CD4^+^ and CD8^+^ cells and were associated with early recurrence in HGSOC [66]. The identification of these ILCregs in ex vivo TIL cultures prompts to the characterization of their counterparts in primary HGSOC tumours. Surface markers and transcription factors that can distinguish CD56^+^ ILCregs from other CD56 expressing ILCs within the tumour remain to be determined. Critically, the interactions of CD56^+^ ILCregs with other immune/stromal cells in the TME need to be further elucidated. 

Several studies have identified CD56^+^ ILCs within both primary tumours [198,199] and ascites [200,201,202,203,204,205,206] of patients with ovarian cancer. Notably, CD56^+^ ILCs from ascites showed reduced expression of activation markers (i.e., DNAM-1, 2B4) [202,204,205], altered metabolic profile [207], and reduced cytolytic capability [204,205,207] compared to their circulating counterparts. However, it is important to note that these earlier studies may have uniformly labelled Lin^−^CD56^+^ as NK cells. We now know that there is incredible heterogeneity within the CD56^+^ population [208,209,210,211] and may include ILC1s [212], ILC3s [213,214,215,216], and ILCregs [66]. Although these studies have attributed these phenotypes to dysfunctional NK cells that are unable to kill tumours, they may represent evidence for non-NK ILCregs. Moreover, ascites from patients with ovarian cancer had a higher proportion of CD56^bright^ CD16^dim^ ILCs compared to peripheral blood [200,202,203,205,206], an ILC subset described as having enhanced suppressive activities against T cells in vitro [68,69,70,72], further suggesting that these subsets are immunoregulatory. Overall, ILCs are beginning to emerge as an important player in immune regulation in cancer. Elucidating the mechanisms of immunoregulatory ILCs will allow for a better understanding of the TME and reveal potential novel targets for patients with ovarian cancer and beyond. 

## 6. Concluding Remarks

Collectively, ILCs have been shown to inhibit immune responses using a variety of mechanisms and have added an additional layer of complexity to our understanding of immune regulation. Although immunosuppressive ILCs mirror suppressive mechanisms as Tregs, they have tissue-resident properties and respond rapidly to immune and physiological signals within the tissue microenvironment. Thus, they are likely to have unique roles in modulating and suppressing innate and adaptive immunity particularly in the context of cancer. One of the major challenges in studying the roles of ILCs in cancer is the inability to easily distinguish immune-suppressive ILCs from immune-promoting ILCs. There are currently no surface marker(s), transcription factor(s), or epigenetic marker(s) that uniquely define immunosuppressive ILCs. Moreover, we currently do not have a strong understanding of where, or what cell-of-origin, they came from. 

It is also possible that some, or all, populations of immunosuppressive ILCs are conventional ILCs that adopt immunosuppressive programs. If this is the case, what are the cytokines and physiological signals that promote immunosuppressive functions in ILCs? Are these immunosuppressive states plastic and reversible? If so, what are the cellular and molecular targets that can be used to manipulate ILCs, shifting their function from immune suppression to promoting anti-tumour immunity? Finally, in the context of human tumours, are there certain types of tumours that are more prone to ILC-mediated immune regulation? Future investigations into these unanswered questions may provide novel insights towards ILC biology and give rise to novel therapeutic targets to enhance anti-tumour immunity. 

## Figures and Tables

**Table 1:** Mechanisms of ILC-mediated immune suppression in cancer.

Cells	Tumour Types	Factors that Promote ILCs	Target Cells	Factors Involved in Suppression	Outcomes	Species	Ref.
**Direct suppression of anti-tumour cytolytic cells**							
NK cells, ILC1s, or CD56^+^ ILCs	Breast & Sarcoma	4-1BBL & STAT3	CD4^+^ T cells	IL-10 & TGF-β	CD73^+^ NK cells suppressed CD4^+^ T-cell proliferation and IFN-γ production through an IL-10 dependent mechanism. The expression of IL-10 and TGF- β by CD73^+^ NK cells was dependent on STAT3 signaling.	*Hu*	[71]
	Ovarian	NKp46 agonist	CD4^+^/CD8^+^ T cells	n/a	CD3⁻CD56^+^ ILCregs from slow growing TIL cultures reduced CD4^+^ and CD8^+^ T cell absolute number and IFN-γ expression.	*M*	[66]
ILC2	Melanoma	n/a	NK cells	CD73	ILC2s suppressed NK cell cytotoxicity and IFN-γ production through a CD73 dependent mechanism.	*M*	[86]
ILC3	Liver	IL-23	CD8^+^ T cells	(Killing)	IL-23 induced ILC1-to-ILC3 conversion. ILC3s directly inhibited CD8^+^ T cell proliferation and increased apoptosis in a cell-to-cell contact dependent manner.	*M*	[134]
**Inhibition of antigen presenting cells**							
NK cells, ILC1s, or CD56^+^ ILCs	Fibrosarcoma	n/a	APCs	PD-L1	NK cells suppressed APC activation and maturation. Presence of NK cells were associated with a reduced antigen-specific memory T cell response to tumour.	*M*	[97]
	Lymphoma	n/a	APCs	n/a	NK cells reduced proliferation of antigen-specific CD8^+^ T cells through CD11c^+^ APCs in the presence of P815 tumour cells.	*M*	[98]
	Mastocytoma & Melanoma	n/a	APCs	TRAIL (Killing)	NK cells regulated APC cross-presentation in the lymph node, reducing proliferation and number of antigen-specific CD8+ T cells.	*M*	[99]
**Enhanced immunosuppressive cells**							
NK cells, ILC1s, or CD56^+^ ILCs	Lung	IL-2	Tregs	CCL22	NK cells secreted CCL22 in tumour cultures. NK1.1^+^ NK cells and FOXP3^+^ Tregs clustered together in LCC tumours.	*M*	[135]
	Prostate	n/a	TAMs	n/a	Cultured media from NK cells induced expression of TAM related genes on monocyte cell lines.	*H*	[136]
ILC2	AML	PGD2	Tregs	IL-5	Mesenchymal stromal cells from AML patients induced IL-5 secretion by ILC2s through PDG2, subsequently enhancing Treg activity.	*Hu/M*	[137]
	APL	B7H6, PGD2	MDSCs	IL-13	PDG2 and B7H6 activated ILC2 secretion of IL-13 to enhance MDSC suppression of T cells.	*Hu/M*	[130]
	NMIBC, MIBC	n/a	MDSCs	IL-13	The proportion of ILC2s was associated with the presence of MDSCs in urine. ILC2s secrete IL-13; IL-13 induces CD14^+^ MDSCs to be more suppressive to T cells	*Hu*	[125]
	NSCLC	n/a	TAMs	IL-4 & IL-13	Cultured media from ILC2s induced CD14^+^ myeloid cells to express M2-like macrophage related markers.	*Hu*	[138]
	Melanoma	n/a	Eosinophils	IL-5	ILC2s reduced IFN-γ expression on NK cells from metastasis in the lung after IL-33 treatment through an IL-5 dependent mechanism. IL-5 may be acting through eosinophils, and was associated with reduced IFN-γ and GZMB expression by NK cells.	*M*	[133]

4-1BBL, 4-1BB ligand; AML, acute myeloid leukemia; APC, antigen-presenting cells; APL, acute promyelocytic leukemia; CCL22, C-C motif chemokine ligand 22; FOXP3, forkhead box P3; GZMB, granzyme B; Hu, human; IFN-γ, interferon γ; IL, interleukin; ILC, innate lymphoid cells; ILCregs, regulatory innate lymphoid cells; LCC, Lewis lung carcinoma; M, mouse; MDSCs, myeloid-derived suppressor cells; MIBC, muscle invasive bladder cancer; NK, natural killer; NMIBC, non-muscleinvasive bladder cancer; NSCLC, non-small cell lung carcinoma; PDG2, prostaglandin D2; STAT3, signal transducer and activator of transcription 3; TAMs, tumour-associated macrophages; TGF-β, transformed growth factor beta; TILs, tumour-infiltrating lymphocytes; TRAIL, TNF-related apoptosis-inducing ligand.
